# Tissue Factor-Enriched Neutrophil Extracellular Traps Promote Immunothrombosis and Disease Progression in Sepsis-Induced Lung Injury

**DOI:** 10.3389/fcimb.2021.677902

**Published:** 2021-07-14

**Authors:** Hao Zhang, Yilu Zhou, Mengdi Qu, Ying Yu, Zhaoyuan Chen, Shuainan Zhu, Kefang Guo, Wankun Chen, Changhong Miao

**Affiliations:** ^1^ Department of Anesthesiology, Zhongshan Hospital, Fudan University, Shanghai, China; ^2^ Cancer Center, Zhongshan Hospital, Fudan University, Shanghai, China; ^3^ Department of Anesthesiology, Shanghai First Maternity and Infant Hospital, Tongji University School of Medicine, Shanghai, China

**Keywords:** tissue factor, neutrophil extracellular traps, immunothrombosis, sepsis, acute lung injury

## Abstract

**Background:**

Patients with sepsis may progress to acute respiratory distress syndrome (ARDS). Evidence of neutrophil extracellular traps (NETs) in sepsis-induced lung injury has been reported. However, the role of circulating NETs in the progression and thrombotic tendency of sepsis-induced lung injury remains elusive. The aim of this study was to investigate the role of tissue factor-enriched NETs in the progression and immunothrombosis of sepsis-induced lung injury.

**Methods:**

Human blood samples and an animal model of sepsis-induced lung injury were used to detect and evaluate NET formation in ARDS patients. Immunofluorescence imaging, ELISA, Western blotting, and qPCR were performed to evaluate *in vitro* NET formation and tissue factor (TF) delivery ability. DNase, an anti-TF antibody, and thrombin inhibitors were applied to evaluate the contribution of thrombin to TF-enriched NET formation and the contribution of TF-enriched NETs to immunothrombosis in ARDS patients.

**Results:**

Significantly increased levels of TF-enriched NETs were observed in ARDS patients and mice. Blockade of NETs in ARDS mice alleviated disease progression, indicating a reduced lung wet/dry ratio and PaO2 level. *In vitro* data demonstrated that thrombin-activated platelets were responsible for increased NET formation and related TF exposure and subsequent immunothrombosis in ARDS patients.

**Conclusion:**

The interaction of thrombin-activated platelets with PMNs in ARDS patients results in local NET formation and delivery of active TF. The notion that NETs represent a mechanism by which PMNs release thrombogenic signals during thrombosis may offer novel therapeutic targets.

## Introduction

Acute lung injury (ALI) and acute respiratory distress syndrome (ARDS) are leading causes of death (30% to 60%) in patients with sepsis (Cecconi et al., 2018; Fan et al., 2018; Matthay et al., 2019). Both ALI and ARDS are characterized by diffuse alveolar damage and increased alveolar capillary permeability (Herrington et al., 2019). Evidence indicates that thrombosis and coagulopathy are major contributors to ALI and ARDS (Helms et al., 2020). However, the underlying mechanism is still unclear.

Neutrophil extracellular traps (NETs) can facilitate pathogen trapping and killing (Brinkmann et al., 2004). They also produce cytotoxic molecules and proteases that cause significant tissue damage (Munoz et al., 2017; Papayannopoulos, 2018). In addition, NETs have been suggested to be key drivers of infection-induced coagulopathy and thrombogenicity in disease models, including sepsis, transfusion-related acute lung injury, deep venous thrombosis, and cancers (Caudrillier et al., 2012; Camicia et al., 2014; Skendros et al., 2020). A recent study further suggested that complement and tissue factor-enriched NETs are key drivers of COVID-19 immunothrombosis formation (Middleton et al., 2020; Stakos et al., 2020), which is a key event in sepsis-induced lung injury. However, few studies have explored whether and how NETs are involved in the pathology of sepsis-induced lung injury.

Tissue factor (TF) can be produced by endothelial cells, smooth muscle cells, neutrophils, and monocytes in response to a variety of stimuli (DelGiudice and White, 2009; Kambas et al., 2012), and it is the major initiator of the coagulation process *in vivo* (Rapaport and Rao, 1995; Mackman et al., 2007). To maintain homeostasis, TF is usually encrypted with very limited function (DelGiudice and White, 2009). Tissue damage and inflammation cause the externalization and activation of TF and the subsequent coagulation (Frantzeskaki et al., 2017). Studies in antineutrophil cytoplasmic antibody (ANCA)-associated vasculitis (AAV) and acute myocardial infarction have demonstrated that polymorphonuclear neutrophils (PMNs) can generate NETs and enhance thrombotic mechanisms by promoting the production of TF (Kambas et al., 2014; Stakos et al., 2015). However, how intracellular TFs in PMNs are externalized and become functional is still not clear. In the culprit artery of acute myocardial infarction and COVID-19 immunothrombosis, NETs were suggested to be vital for active TF delivery (Stakos et al., 2015; Skendros et al., 2020), indicating NETs as a potential mechanism for TF externalization during infection. Several mechanisms by which PMNs and their production of TF trigger thrombotic events have been reported (Ritis et al., 2006; Egorina et al., 2008; Kambas et al., 2012), although how this event occurs in sepsis-induced lung injury is not yet clear.

Using human samples and a mouse model of sepsis-induced lung injury, we found that NETs were closely related to disease progression. Moreover, increased TF production and exposure to NETs were observed in ARDS patients. An *in vitro* model further demonstrated that thrombin-activated platelets were responsible for increased TF-enriched NET formation and subsequent immunothrombosis, which may worsen the progression of sepsis-induced lung injury.

## Materials and Methods

### Study Population

The study complied with the Declaration of Helsinki and was approved by the Ethics Committee of Fudan University Shanghai Cancer Center (license number: 20180109-04). Written informed consent was obtained from all subjects. Patients admitted to the intensive care unit (ICU) from January 2018 to December 2018 were enrolled in the study. The inclusion criteria included: ages between 18 and 70 years, diagnosed with sepsis or sepsis-induced ARDS (ARDS) after surgery, and an expected ICU hospitalization longer than 24 h. The exclusion criteria were as follows: history of cardiopulmonary arrest before ICU admission; history of connective tissue disorders, such as vasculitis; pregnancy; and any history of vascular embolism. Patients were diagnosed with sepsis according to the third international consensus definition for sepsis (Singer et al., 2016). ARDS was diagnosed in patients with severe sepsis or septic shock receiving mechanical ventilation (MV) for hypoxemic respiratory failure, and they met the diagnostic criteria for acute lung injury-ARDS according to the 2012 Berlin criteria for the diagnosis of ARDS (Fan et al., 2018).

### Data Collection and Blood Sample Measurements

Demographic data were recorded from patients upon admission to the ICU. All patients received computed tomography (CT) to assess the severity of lung injury upon admission to the ICU. The PaO_2_/FiO_2_ ratio was also calculated at this time. The Acute Physiology and Chronic Health Status Scoring System II (APACHE II) score (Knaus et al., 1985) was calculated to determine the severity of sepsis.

Ten milliliters of peripheral venous blood were taken within 1 h of ICU admission. Peripheral blood neutrophil counts, lymphocyte counts, monocyte counts, platelet counts, hemoglobin concentration (g/dl), erythrocyte sedimentation rate (ESR), C-reactive protein (CRP) levels, albumin concentration activated partial thromboplastin time (APTT, in seconds), prothrombin time (PT, in seconds), fibrinogen, and D-dimer concentrations were obtained and recorded. Interleukin (IL)-6 (pg/ml) and IL-8 levels (pg/ml) were measured by ELISA. Morning fasting peripheral venous blood was taken from healthy controls (HCs).

### Human Neutrophil Isolation

In our study, human PMNs were isolated as previously reported (Brinkmann et al., 2010). Briefly, 10 ml of human blood was prepared with heparin (10 U/ml). Five milliliters of Histopaque1119 (Sigma-Aldrich) were added to a 15-ml Falcon tube, and 5 ml of whole blood was placed on the top. Cells were centrifuged at 800*g* for 30 min, and the yellowish phase was discarded. Meanwhile, the lower reddish phase containing granulocytes was transferred into Falcon tubes. The cells were washed with PBS and centrifuged at 300*g* for 15 min. At the same time, a 100% Percoll solution was prepared by mixing 18 ml Percoll with 2 ml 10× PBS. After centrifugation, the pellets were combined, and the sedimented cells were resuspended in 5 ml of PBS. Two ml of resuspension was layered onto the gradients and centrifuged at 800*g* for 20 min. After centrifugation, the top layer was removed, and the remaining white interface was collected into Falcon tubes. The Falcon tubes were washed with PBS and centrifuged at 300*g* for 10 min. Finally, sedimented cells were resuspended in 2 ml of PBS (with 95% purity).

### Animal Model of Sepsis-Induced Lung Injury

C57BL/6J mice aged 6 to 8 weeks were obtained from the Animal Research Center of Fudan University (Shanghai, China). All animal experiments were conducted in accordance with the relevant guidelines and regulations approved by the institutional animal care and use committee of Fudan University. An experimental cecal ligation and puncture (CLP) sepsis mouse model was used (Rittirsch et al., 2009). Briefly, SPF C57BL/6J mice weighing 25 to 30 g were anesthetized by intraperitoneal injection of 1% pentobarbital sodium (1 mg/kg). The abdomen was disinfected with 70% ethanol, a 1- to 2-cm-long incision was made along the middle of the abdomen, and the abdominal cavity was opened. Then, the cecum was exposed, ligated with a 5-0 suture, and punctured using a 20-gauge needle. Once needle-sized feces were detected, the cecum was placed back to its original position, and the abdomen was closed. All animals received 0.5 ml/10 g of normal saline for rehydration. After surgery, the animals were placed in their cage and monitored every two hours until sampling. Control mice referred to healthy controls. Sham mice underwent the same surgical procedures without cecal puncture or ligation.

### Lung Histology and Wet-to-Dry Weight Ratio

Lungs were fixed in 10% neutral formalin solution for 24 h. Then, sections of the lungs were embedded in conventional paraffin and stained with hematoxylin and eosin. A semiquantitative scoring system was used to evaluate the severity of lung injury. The system evaluates four items, including alveolar congestion and hemorrhage, alveolar wall/hyaline membrane formation, neutrophil infiltration or aggregation in alveoli or blood vessels, and the degree of inflammatory cell infiltration (Randrianarison et al., 2008). Two pathologists blinded to the results scored the specimens. Specimens were scored as follows: 0, no injury; 1, mild injury (25%); 2, moderate injury (50%); 3, severe injury (75%); and 4, very severe injury (almost 100%) for each item. Then, each item was added for a possible maximum of 16 points (highest severity) (Uzunhan et al., 2016).

The wet-to-dry weight ratio was calculated to evaluate the degree of edema of the lung tissue (Planes et al., 2010). For wet/dry ratio calculations, the surface water of the lung tissue was absorbed with filter paper and placed in a clean and dry glass test tube to obtain the wet weight of the specimen. Then, each specimen was desiccated at 70°C for 48 h. Finally, the PaO_2_/Fio_2_ was calculated before euthanasia.

### Stimulation and Inhibition Studies

PMNs from healthy controls were initially cultured in RPMI (Gibco BRL) mixed with 5% FBS (Gibco BRL). For *in vitro* studies, PMNs isolated from healthy controls were treated for a duration of 6 h with plasma or platelets from healthy controls, patients with sepsis, or patients diagnosed with sepsis-ARDS. Platelets isolated from HC were treated with plasma from HC, sepsis, and sepsis-induced ARDS for 30 min at 37°C for platelet activation. Platelets were also cocultured with plasma from the three groups to induce NET formation.

For the two-procedure stimulation of PMNs, platelets were first pretreated with plasma from ARDS for 30 min to induce their activation. Then, activated platelets were cocultured with PMNs from healthy controls and plasma from ARDS patients. Platelets were pretreated with FLLRN peptide (500 mM, Anaspec) for 30 min to block protease-activated receptor 1 (PAR-1) signaling (Holinstat et al., 2011). ARDS plasma was treated with antithrombin III (Sigma-Aldrich, SML2370) or dabigatran (Sigma-Aldrich, A2221) for 30 min for thrombin inhibition. Recombinant thrombin (0.01 U, Calbiochem) was used as a positive control for healthy control platelet activation. For the combined stimulation, dabigatran was used in both ARDS plasma for platelet activation and subsequent PMN stimulation.

To explore the impact of neutrophil depletion and NET degradation on the progression of sepsis-induced lung injury, DNase (Sigma-Aldrich, D5025), NE inhibitor (ab142369), anti-Ly6G (Abcam, ab238132), and Cl-amidine (Sigma-Aldrich, 506282) were used as previously described (Williams et al., 2001; Cummins et al., 2008). Briefly, mice were injected intravenously with 65 U DNase (Sigma-Aldrich) in 100 μl 0.9% sodium chloride solution 6 h after cecal puncture. For human samples, DNase was added after PMNs were cultured. NET degradation is being induced through DNase treatment in the experiment. NETs were identified as structures positive for citH3. NET degradation in peripheral neutrophils was quantified by examining 100 cells in a double-blind experimental procedure.

### NET Immunofluorescence

Pulmonary blood vessels from ARDS mice were carefully separated, and the *in situ* thrombus attached to the inner wall of the blood vessel was detected under a microscope. The *in situ* thrombus was then carefully detached and fixed in 4% formaldehyde solution. Then, immunofluorescence staining was performed to observe NET formation. NET analysis was performed by immunofluorescence using confocal microscopy as previously reported (Barnes et al., 2020; Hisada et al., 2020). NETs were stained with anti-myeloperoxidase (diluted 1:100; Abcam 134132), anti-histone H3 (diluted 1:100, citrulline R2+R8+R17; Abcam 5103), anti-neutrophil elastase (diluted 1:100; Abcam 254178), anti-Ly6G (diluted 1:100, ab238132), and anti-tissue factor (diluted 1:100, Abcam 228968) antibodies. Alexa Fluor 488 (1:200, Life Technologies A-21206) and Alexa Fluor 555 (1:200, Life Technologies A-21432) were used as secondary antibodies. DNA was stained with DAPI (Sigma-Aldrich). The percentage of NET-releasing cells was calculated by examining 100 cells in a double-blind experimental procedure (Nie et al., 2019).

### Western Blot Analysis

Immunoblotting for TF determination was performed as previously described (Eriksson et al., 2016). Briefly, total protein was extracted from samples, and the protein concentration was measured using BCA protein assay reagent (Beyotime, Shanghai, China). Then, the extracted protein was separated by sodium dodecyl sulfate polyacrylamide gel electrophoresis and detected by immunoblotting with specific antibodies against tissue factor (Abcam, ab228968).

### Quantitative PCR

Total RNA was extracted by TRIzol reagent (Invitrogen, MA, USA), and cDNA was synthesized with a Transcriptor First Strand cDNA Synthesis Kit (Roche, Basel, Switzerland) according to the manufacturer’s instructions. Primers specific for mouse TF mRNA were detected using forward primers spanning exons 4 and 5 (5′-TCAAGCACGGGAAAGAAAAC-3′) and reverse primers located within exon 5 (5′-CTGCTTCCTGGGCTATTTTG-3′).

### Flow Cytometry Analysis

Platelets were stained with anti-annexin/ANXA5 antibody (Abcam, ab63556) and anti-CD62P antibody (Abcam, ab234221). Anti-CD61-PerCP antibody (BD Biosciences, 347407) was chosen as the platelet-specific marker, and CD61-CD11b positivity was used as a marker of PMN/platelet aggregation. For neutrophils, CD11b (Abcam, 133357) was used as a specific marker. PMNs were identified as CD11b-positive events (Yoon et al., 2007; Adan et al., 2017; Kang et al., 2018).

### Myeloperoxidase-DNA Complex ELISA Measurement

MPO-DNA complexes were used to quantify NET release *in vivo* and *in vitro*. MPO-DNA complexes were quantified by capture ELISA as previously described (Aldabbous et al., 2016; Petersen et al., 2018).

### MPO Activity Measurements

A myeloperoxidase activity assay (ab105136) was used to test MPO activity. MPO activity was quantified as previously described (Huang et al., 2016).

### Quantification of Cell-Free DNA

Cell-free DNA (cf-DNA) in serum and culture supernatant was detected by a Quant-iTPicoGreen^®^ dsDNA kit (Invitrogen) according to the manufacturer’s instructions. The amount of DNA was measured by the fluorescence intensity (480 and 530 nm).

### Thrombin–Antithrombin Complex ELISA

Thrombin concentration was measured in *ex vivo* plasma obtained from the blood of HC, sepsis, and ARDS patients by a TAT complex ELISA kit (Abcam, ab108907) according to the manufacturer’s instructions (Zhao et al., 2017).

### Statistical Analysis

Patient demographics and treatments were compared using a chi-square test or Student’s *t* test. Survival data were calculated by the Kaplan-Meier method and compared with the log-rank test. Correlations were analyzed by means of Spearman’s or Pearson’s tests. NET cutoff points were generated and analyzed using X-tile 3.6.1 software (Yale University, New Haven, CT, USA), which identified the cutoff with the minimum P values from log-rank ×2 statistics for survival (Camp et al., 2004). All laboratory experiments were performed in triplicate. A P value <0.05 was considered statistically significant. Statistical analyses were performed with SPSS 20.0 (SPSS Inc. Chicago, IL, USA).

## Results

### Higher Neutrophil Extracellular Trap Levels Correlate With Worse Survival in Sepsis-Induced ARDS Patients

**Figure 1:**
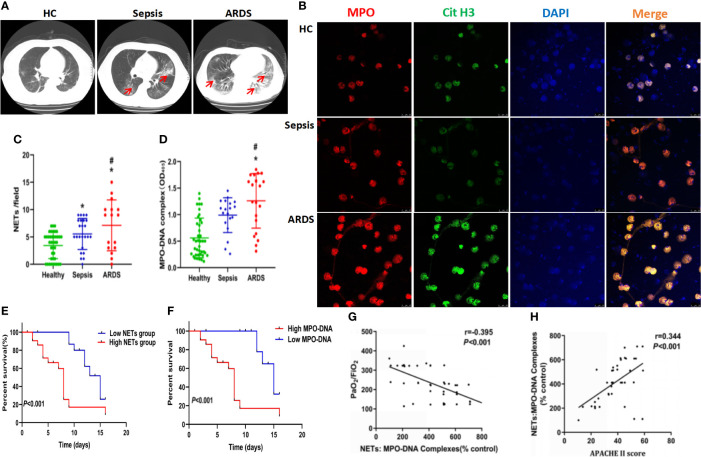
High levels of neutrophil and neutrophil extracellular traps accumulate in sepsis ARDS patients and correlate with worse outcomes. **(A)** CT imaging of the lungs of healthy volunteers, sepsis patients, and ARDS patients. **(B)** Fluorescence imaging of human peripheral blood neutrophils among healthy volunteers, sepsis patients, and ARDS patients for 6 hours. The sections were immunostained with MPO (red) and CitH3 (green), and DAPI (blue) was used to counterstain the nuclei. **(C)** *Ex vivo* NET assay of neutrophils in healthy volunteers, sepsis patients, and ARDS patients. **(D)** *Ex vivo* MPO-DNA complex ELISA in healthy volunteers, sepsis patients, and ARDS patients. **(E)** Patient survival curve in the low or high NET group of ARDS patients (n=16). **(F)** Patient survival curve in the low or high MPO-DNA group. **(G)** Correlation curve between the MPO-DNA complex and PaO_2_/FiO_2_. **(H)** Correlation curve between APACHE II score and MPO-DNA complex. Statistical analysis was performed using the log-rank (Mantel-Cox) test. P < 0.05 (*) and P < 0.05 (^#^) compared to baseline healthy controls and sepsis patients were considered statistically significant.

Twenty-four and 16 patients diagnosed with sepsis and ARDS, respectively, were included in the study. The healthy control group consisted of 40 age-matched volunteers. More severe lung damage was observed in patients with ARDS than in the healthy control and sepsis groups (**Figure 1A**), which was accompanied by higher neutrophil numbers and higher serum levels of the inflammatory cytokines IL-6 and IL-8 in ARDS patients (**Table 1**). To explore whether NETs were associated with lung damage in ARDS patients, we detected NET levels in peripheral blood by immunostaining neutrophils with MPO and CitH3, which are biomarkers of NET formation. Compared with patients from the healthy control and sepsis groups, higher levels of NETs per field in the blood of ARDS patients were observed [7 (3–13) *vs.* 3 (1-5), P<0.001, 7 (3-13) *vs.* 4 (3-7), P=0.023, respectively, **Figures 1B, C**]. As expected, the serum level of MPO-DNA complexes was significantly increased in ARDS patients compared with healthy controls and sepsis patients [1.3 (0.6-1.7) *vs.* 0.6 (0.2-0.9), P<0.001, 1.3 (0.6-1.7) *vs.* 1.0 (0.7-1.2), P=0.011, respectively, **Figure 1D**]. High NET levels, with four NETs per field as the cutoff value, and high MPO-DNA levels were further found to be associated with worse survival in ARDS patients in our study (**Figures 1E, F** **)**. Our data also showed a strong correlation of MPO-DNA complex levels with PaO_2_/Fio_2_ and APACHE II scores (r=-0.395, P<0.001, r=0.344, P<0.001, respectively) (**Figures 1G, H** **)**. Overall, the high NET levels and related poor survival in ARDS patients in our study indicate a potential role of NETs in the pathology of ARDS.

**Table 1:** Baseline of characteristics of healthy and patients enrolled in the group.

	Healthy Control (N = 40)	Sepsis (N = 24)	Sepsis ARDS (N=16)
**Gender (male, %)**	24 (60%)	12 (50%)	9 (56%)
**Ages (years), mean±SD**	45.6±14.2	44.4±13.5	45.9±13.6
**BMI (kg/m^2^), mean±SD**	28.6±6.5	27.6±6.4	27.2±6.3
**Neutrophils (10^9^/L)**	3.2±0.8	5.0±1.4*	6.8±3.2*^#^
**Monocytes (10^9^/L)**	0.3±0.2	0.7±0.3	0.8±0.2
**Lymphocytes (10^9^/L)**	1.9±0.3	1.5±0.5	1.4±0.4
**Platelets (10^9^/L)**	230.9±38.4	346.7±105.4*	390.5±179.4*^#^
**Hemoglobin (g/L)**	148.2±12.5	120.3±27.5*	100.6±22.1*^#^
**ESR (mm/h)**	8.2±2.1	28.5±3.4*	48.9±21.5*^#^
**CRP (mg/L)**	4.1±0.5	75.1±30.2*	125.3±40.2*^#^
**Albumin(g/L)**	46.2±2.5	36.2±6.1*	31.5±6.8*^#^
**PT(s)**	11.1±1.0	10.5±1.4	10.8±1.5
**APTT(s)**	32.4±3.2	33.4±5.4	35.4±4.3
**D-dimer(ng/ml)**	90±69.4	195.4±91.5*	326.5±100.2*^#^
**Fibrinogen(g/L)**	2.8±0.4	4.2±1.0*	6.2±1.2*^#^
**Plasma cytokine level(pg/ml), median (IQR)**			
**IL-6**	27 (25-32)	620 (530-670)*	850 (680-1070)*^#^
**IL-8**	51 (41-67)	380 (310-490)*	530 (410-620)*^#^
**PaO2/FiO2**	410±26	330±30*	260±25*^#^

BMI, body mass index; ESR, erythrocyte sedimentation rate; IQR, interquartile range; CRP, C reactive protein.

Data are expressed by percentage or median (interquartile range [IQR]), mean ± standard deviation [SD], *P < 0.01 versus controls; **^#^**P < 0.01 versus sepsis patients.

Wh612699.

### NET Inhibition Protects Mice Against Sepsis-Induced Lung Injury

**Figure 2:**
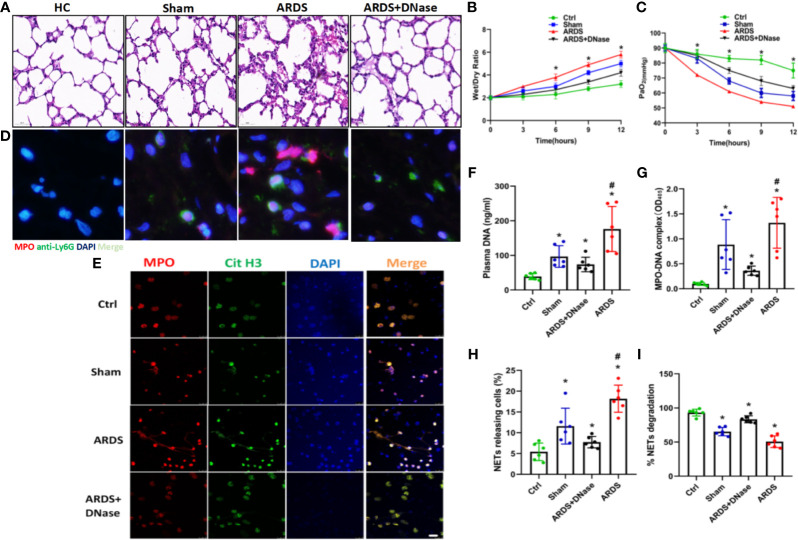
NETs increase in a sepsis-induced lung injury model. **(A)** Lung tissues were collected from C57BL/6 mice in the control group, sham group, ARDS group, or ARDS+DNase group. H&E staining was performed on paraffin-embedded sections of mouse lungs to detect lung injury. **(B)** Changes in the lung wet/dry ratio in the four groups **(C)**. Changes in PaO_2_ as the processing time increased in the four groups. **(D)** Immunofluorescence of lung tissue in red (MPO) and blue (DAPI). **(E)** Fluorescence imaging of mouse peripheral blood neutrophils among the healthy control, sham, ARDS, and ARDS+DNase groups. The sections were immunostained with MPO (red) and CitH3 (green), and DAPI (blue) was used to counterstain the nuclei. **(F)**. *Ex vivo* plasma DNA. **(G)** MPO-DNA complex. **(H)** NET-releasing cell count assay. **(I)** NET degradation among the healthy control, sham, ARDS, and ARDS+DNase groups (n=6). P < 0.05 (*) and P < 0.05 (^#^) compared to baseline healthy controls and the sepsis group were considered statistically significant.

To further explore the role of NETs in ARDS, a mouse model of sepsis-induced lung injury was established, and healthy control and sham mice were compared. Compared with control and sham mice, specimens from ARDS mice showed significantly increased lung injury, as defined by diffuse disruption of the alveolar wall and massive inflammatory cell infiltration, which could be significantly alleviated by DNase treatment (**Figure 2A** and **Supplementary 1G**). As expected, an increase in the lung wet/dry weight ratio was observed in ARDS mice compared with control and sham mice at 6, 9, and 12 h after model establishment (**Figure 2B**). A decrease in PaO_2_ was observed in ARDS mice compared with control and sham mice at 3, 6, 9, and 12 h after model establishment (**Figure 2C**). When ARDS mice were treated with DNase, their increased lung wet/dry weight ratio and decreased PaO_2_ were partially reversed (**Figures 2B, C** **)**.

To further check the level of NETs in ARDS mice, biomarkers of NETs and NETosis were detected. We observed significantly elevated levels of NETs in the lung tissues (**Figure 2D**, **Supplementary Figures 1E, F, H**), MPO/CitH3 expression in peripheral blood neutrophils (**Figure 2E**), cell-free plasma DNA (**Figure 2F**), and serum MPO-DNA complexes (**Figure 2G**) and a significantly increased number of cells releasing NETs (**Figure 2H**). Increased NET levels, indicated by elevated biomarkers mentioned above, were then significantly decreased by DNase treatment (**Figures 2F–I**). Higher NET levels are usually associated with impaired NET degradation. We, therefore, compared NET degradation among control, sham, ARDS, and ARDS mice treated with DNase. Our results showed significantly decreased NET degradation in ARDS mice, which could be reversed by DNase treatment (**Figure 2I**). Anti-Ly6G antibody and PAD4 inhibitor CI-amidine, known to deplete neutrophils, were also found to significantly inhibit NETs levels in ARDS mice as indicated by reduced serum levels of cell-free DNA (**Supplementary Figure 1A**), MPO (**Supplementary Figure 1B**), and MPO-DNA complexes (**Supplementary Figure 1C**) as well as reduced expression of CitH3 in lung tissues of ARDS mice after treatment (**Supplementary Figure 1D**). We also stained NETs with neutrophil elastase (NE) and NE inhibitor to detect NET formation in different groups. Additionally, MPO activity was assessed in each group. In summary, the findings shown above suggest a potential role for NET inhibition in protecting against sepsis-induced lung injury (**Supplementary Figures 2A–C**).

### TF-Enriched NETs in Polymorphonuclear Neutrophils Contribute to Immunothrombosis in ARDS Patients

**Figure 3:**
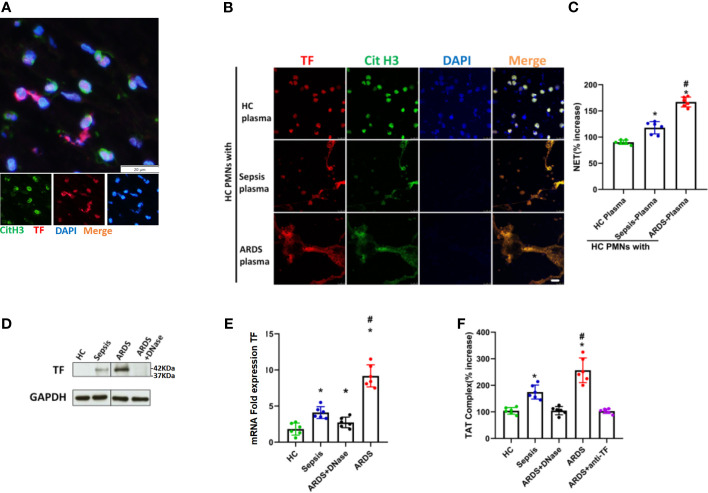
Intracellular TF in polymorphonuclear neutrophils of ARDS patients is associated with NET formation. **(A)** Fluorescence imaging of lung thrombi in ARDS mice. The sections were immunostained with TF (red) and CitH3 (green), and DAPI (blue) was used to counterstain nuclei. **(B)** Plasma of healthy controls (HC), sepsis or ARDS patients was cocultured with polymorphonuclear neutrophils (PMNs) in HC. Fluorescence imaging of PMNs in the three groups. The sections were immunostained with TF (red) and CitH3 (green), and DAPI (blue) was used to counterstain the nuclei. **(C)** An increase in NETs was detected by coculture with different plasma samples (n=6). **(D)** Western blots for TF expression in the HC, sepsis, ARDS or ARDS+DNase groups (n=6). **(E)** mRNA fold change for TF expression in HC, sepsis, ARDS or ARDS+DNase groups (n=6). **(F)** TAT complex progression in the HC, sepsis, ARDS+DNase, ARDS, or ARDS+ anti-TF groups (n=6). P < 0.05 (*) and P < 0.05 (^#^) compared to baseline healthy controls and the sepsis group were considered statistically significant.

TF, as one of the primary initiators of blood coagulation, has been shown to be closely associated with NET formation. To check the expression and distribution of TF in ARDS mice, lung thrombi of ARDS mice were isolated, and the expression of TF and CitH3 was detected. Fluorescence imaging showed clear exposure of NETs to TF, as indicated by the coexpression of TF and CitH3 in lung thrombi (**Figure 3A**). To explore factors causing the increased TF level and its exposure to NETs, neutrophils from healthy controls were cocultured with plasma from healthy controls, sepsis patients, and ARDS patients. TF and CitH3 expressions were detected on neutrophils to determine TF/CitH3 colocalization. We observed massive NET formation in neutrophils cultured with ARDS plasma (**Figures 3B, C** **)**, in which TF expression by neutrophils was significantly increased, and NETs were exposed (**Figure 3B**). Enhanced TF expression at both the protein and mRNA levels in the ARDS plasma culture group was also shown through Western blot and qPCR analyses (**Figures 3D, E** **)**. Significantly decreased TF expression upon DNase treatment (**Figures 3D, E** **)** further demonstrated increased TF in the ARDS group exposed to NETs. The thrombin–antithrombin (TAT) complex, considered one of the biomarkers of immunothrombosis, was then detected by ELISA. Significantly enhanced TAT complex levels were found in the ARDS group (**Figure 3F**). Moreover, when treated with DNase or the anti-TF, the enhanced TAT was fully reversed (**Figure 3F**). Overall, the findings described above demonstrated that the plasma of ARDS patients contributes to TF-enriched NET formation, which plays a key role in the immunotherapy of ARDS patients.

### Activated Platelets Induce TF-Enriched NET Formation in ARDS Patients

**Figure 4:**
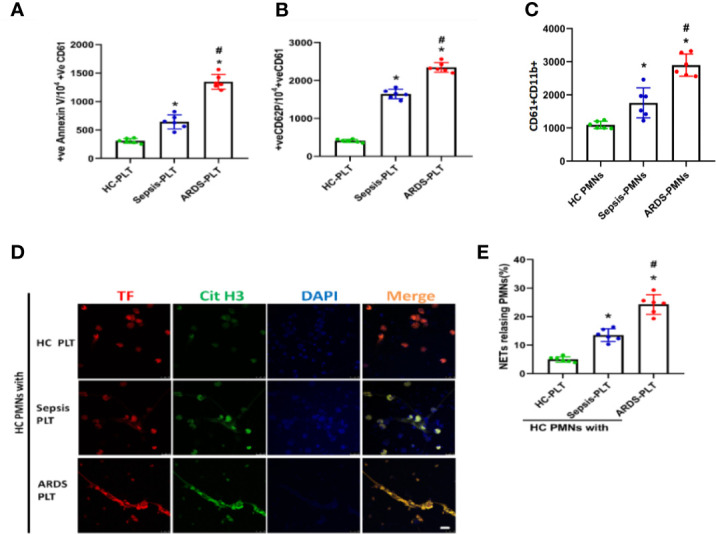
Platelets activated in ARDS are able to induce NET formation. **(A)** Fluorescence-activated cell sorting analysis of Annexin V on platelets of HC platelets, sepsis patients, and ARDS patients (n=6). **(B)** Fluorescence-activated cell sorting analysis of CD62P on platelets of HC platelets, sepsis patients, and ARDS patients (n=6). **(C)** Platelet/polymorphonuclear neutrophil aggregates observed as double-positive CD61/CD11b per 10000 CD11b-positive events with fluorescence-activated cell sorting analysis in polymorphonuclear neutrophils isolated from HC, sepsis, and ARDS patients (n=6). **(D)** NET generation by control polymorphonuclear neutrophils treated with platelets isolated from the 3 groups. The sections were immunostained with TF (red) and CitH3 (green), and DAPI (blue) was used to counterstain the nuclei. **(E)** Percentage of NET-releasing PMNs (n=6). P < 0.05 (*) and P < 0.05 (^#^) compared to baseline healthy controls and the sepsis group were considered statistically significant.

Platelets, as one of the key initiators of thrombosis, have been reported to contribute to NET formation (Caudrillier et al., 2012). We compared the activation status of platelets from different patients and found that platelets from ARDS patients showed increased levels of surface activation markers compared with platelets from sepsis patients or healthy controls (**Figures 4A, B** and **Supplementary Figures 3**, **4**). We then continued to explore the contribution of platelets to the pathology of sepsis-induced lung injury. First, we observed a significant increase in the number of platelet/neutrophil aggregates (CD61+CD11b+) in PMNs isolated from ARDS patients compared to sepsis patients or healthy controls (**Figure 4C** and **Supplementary Figure 5**). To explore whether activated platelets contribute to NET formation and TF exposure on NETs in ARDS patients, neutrophils from healthy controls were cocultured with platelets from healthy controls, sepsis patients, and ARDS patients. Then, TF and CitH3 expression was detected to evaluate the TF-enriched NETs of different culture groups. As expected, neutrophils cultured with ARDS patient-derived platelets showed significantly increased NET release (**Figures 4D, E** **)**.

### Role of ARDS Plasma and Platelets in the Formation of TF-Enriched NETs

**Figure 5:**
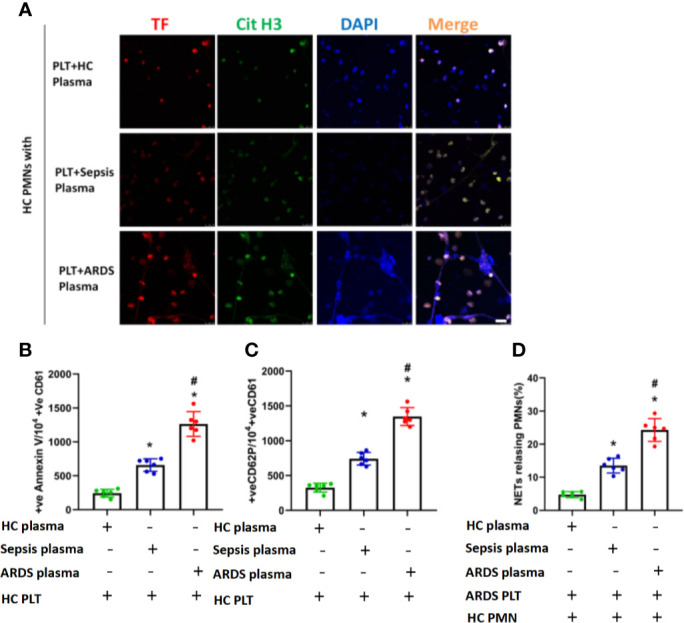
Activated platelets for subsequent NET generation in PMNs. **(A)** Neutrophil extracellular trap formation by control polymorphonuclear neutrophils incubated with control platelets pretreated with plasma obtained from the 3 groups. **(B)** Fluorescence-activated cell sorting analysis of Annexin V on control platelets treated with plasma obtained from the 3 groups (n=6). **(C)** Fluorescence-activated cell sorting analysis of CD62P on control platelets treated with plasma obtained from the 3 groups (n=6). **(D)** Percentage of NET-releasing polymorphonuclear neutrophils of control polymorphonuclear neutrophils treated with platelets and plasma obtained from the 3 groups (n=6). P < 0.05 (*) and P < 0.05 (^#^) compared to baseline healthy controls and the sepsis group were considered statistically significant.

The above findings suggest that both plasma and platelets of ARDS patients contribute to TF-enriched NET formation, which subsequently induces immunothrombosis in ARDS patients. We then continued to confirm the complementary effects of plasma and platelets on the formation of TF-enriched NETs. PMNs from healthy controls were first stimulated with plasma from ARDS patients to induce NET formation, with plasma from healthy controls and sepsis patients as controls. Six hours later, activated platelets from ARDS patients were added to the culture system. Fluorescence imaging of cells from different culture groups was compared. We observed significantly increased NET formation (**Figure 5A**) and numbers of PMNs releasing NETs (**Figure 5D**). We next asked whether plasma from ARDS patients was able to induce platelet activation. Platelets from healthy participants were isolated and stimulated with plasma from healthy controls, sepsis patients, and ARDS patients. As expected, a higher activation level was observed on platelets stimulated with plasma from ARDS patients compared to plasma from healthy or sepsis controls (**Figures 5B, C** **)**. In summary, factors in plasma contribute to the activation of platelets, which play a key role in TF-enriched NET formation and the subsequent immunothrombosis of ARDS patients.

### Thrombin in Plasma from ARDS Is Responsible for Platelet Activation and Subsequent TF-Enriched NET Formation

**Figure 6:**
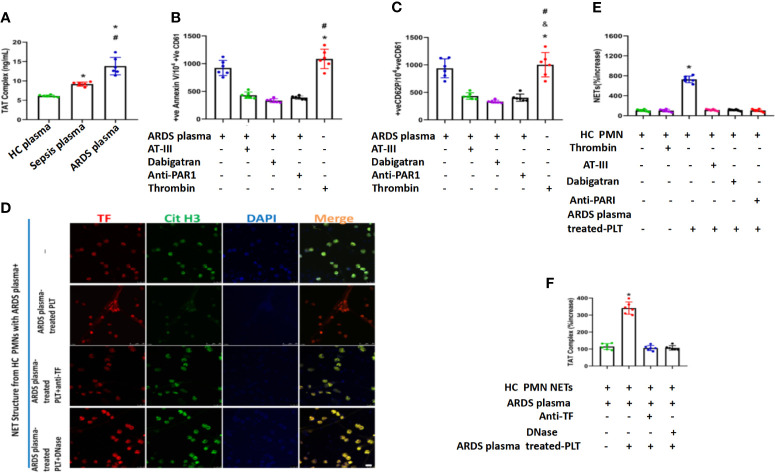
Thrombin in plasma from ARDS is responsible for platelet activation and subsequent NET generation. **(A)** Thrombin levels in plasma obtained from 3 different groups as assessed by TAT complex enzyme-linked immunosorbent assay. Fluorescence-activated cell sorting analysis of Annexin V **(B)** and CD62P **(C)** on control platelets treated with plasma from ARDS patients in the presence or absence of thrombin (antithrombin III or dabigatran) and PAR-1 signaling inhibitors. Recombinant thrombin was used as a positive control (n=6). **(D)** Thrombin levels in control plasma incubated with isolated NET structures from control PMNs stimulated as in ARDS plasma- and ARDS plasma-treated PLTs. **(E)** The NET increase was tested with a myeloperoxidase-deoxyribonucleic acid complex enzyme-linked immunosorbent assay derived after stimulation of control PMNs with plasma from ARDS and control platelets pretreated with ARDS in the presence or absence of thrombin inhibitors (antithrombin III or dabigatran) (n=6). **(F)** Anti-tissue factor antibody was used to neutralize tissue factor-mediated thrombin generation. DNase I was used for neutrophil extracellular trap scaffold degradation (n=6). P < 0.05 (*), P < 0.05 (^#^) and P < 0.01 (^&^) compared to baseline healthy controls and the sepsis group or ARDS group were considered statistically significant.

Thrombin was reported to be necessary for platelet activation through protease-activated receptor-1 (PAR-1) (Petzold et al., 2020). We therefore explored whether thrombin is the factor in plasma that induces platelet activation in ARDS patients. We first detected thrombin levels in the plasma of ARDS patients. Compared with healthy volunteers or sepsis patients, patients with ARDS had significantly elevated thrombin levels (**Figure 6A**). When extra thrombin was added to platelets stimulated with plasma from ARDS patients, a higher activation level of those platelets was observed (**Figures 6B, C** **)**. When thrombin was inhibited using thrombin inhibitors, such as antithrombin III or dabigatran and specific PAR-1 antagonism, the activation of platelets was significantly inhibited (**Figures 6B, C** **)**, suggesting a key role for thrombin in the activation of platelets by ARDS plasma. When PMNs from healthy controls were stimulated with ARDS plasma and ARDS plasma-treated platelets, robust TF-enriched NETs were induced (**Figure 6D**), which were then significantly inhibited when thrombin inhibitors were added to the cultures (**Figure 6E**). PMNs stimulated with both ARDS plasma and ARDS-activated platelets also induced significantly higher TAT levels, which were fully inhibited upon treatment with either TF blocking antibody or DNase (**Figure 6F**), indicating an alleviation of immunothrombosis. Overall, our findings demonstrate that thrombin in plasma in the ARDS environment plays a key role in platelet activation and the subsequent TF-enriched NET induction and immunothrombosis of ARDS patients.

## Discussion

Our studies found significantly elevated levels of TF-enriched NETs in patients with sepsis-induced ARDS, which were demonstrated to contribute to immunothrombosis and worsened disease progression through *in vitro* and *in vivo* models. Fluorescence imaging showed enhanced TF exposure on NETs in ARDS patients, and both the anti-TF antibody and DNase inhibited the formation of TF-enriched NETs and immunothrombosis, as indicated by decreased TAT levels. Our *in vitro* coculture experiment further demonstrated that thrombin-activated platelets were responsible for increased TF-enriched NET formation and subsequent immunothrombosis in ARDS patients, suggesting complementary roles for plasma and platelets in NET-mediated immunothrombosis.

Sepsis can cause damage to multiple organs, among which the lung is the most vulnerable and severely damaged. When sepsis continues to develop, ALI/ARDS will occur, which is one of the leading causes of death in patients.

NETs are released by neutrophils as a novel immune response to infection, and their formation has been reported in a variety of tissues demonstrating their tissue damaging roles (Munoz et al., 2017; Wang, 2018). In addition to fighting against bacterial infection, abnormal NET function has also been reported in a variety of other inflammatory diseases, such as diabetes and cardiovascular diseases (Ravindran et al., 2019); cancers (Acuff et al., 2006; Houghton et al., 2010; Cools-Lartigue et al., 2013; Yang et al., 2020); and immunodeficiency and autoimmune diseases, such as chronic granulomatous disease (CGD), systemic lupus erythematosus (SLE) and rheumatoid arthritis (RA), Behcet’s disease, and vasculitis (specifically anti-neutrophil cytoplasmic autoantibody-associated vasculitis) (Apel et al., 2018; Podolska et al., 2018; Le Joncour et al., 2019). Inducers of NET formation *in vivo* include bacteria, fungal hyphae, biochemical stimuli, some inflammatory cytokines and chemokines (such as TNFα, IL-1β, IL-6, IL-18, and IL-8), immune complexes, and activated platelets. As a key initiator of thrombosis (Denis and Wagner, 2007), platelets are considered potent inducers of NETs (Kim and Jenne, 2016). In transfusion-related acute lung injury, activated platelets can induce NET formation (Caudrillier et al., 2012). In recent COVID-19 pathology, activated platelets from patients were shown to generate TF-bearing NETs, which then induced thrombotic activity of human aortic endothelial cells (HAECs) (Skendros et al., 2020). Vraedon McDonald et al. also reported that platelets and NETs collaborate to promote intravascular coagulation during sepsis in mice (McDonald et al., 2017).

An imbalance between coagulation and inflammation leads to immunothrombosis. Supported by immune cells, platelets, and coagulation-related molecules, immunothrombosis is considered a key event in ARDS pathophysiology (Frantzeskaki et al., 2017). Lines of evidence have reported the contributions of NETs to immunothrombosis. NETs have been reported to be linked to venous thrombosis in breast and pancreatic tumors (Snoderly et al., 2019; Hisada et al., 2020). In a deep vein thrombosis (DVT) model, it was demonstrated that extracellular NETs provide a stimulus and scaffold for thrombus formation (Fuchs et al., 2010). TF-enriched NETs are considered key drivers of COVID-19 immunothrombosis (Skendros et al., 2020).

TF is a membrane protein that plays a critical role in the activation of coagulation (Frantzeskaki et al., 2017). The intracellular localization of TF in neutrophils under normal conditions limits its function (DelGiudice and White, 2009), therefore protecting the host against coagulation activation. TF is activated and becomes functional once it is delivered to the cell membrane (Sebag et al., 2011). Studies have suggested *de novo* TF production by neutrophils in response to inflammatory stimuli (Kambas et al., 2012), while few studies have explored how neutrophils can externalize TF in a functional manner in sepsis-induced lung injury. In COVID-19 immunothrombosis, neutrophils of patients produce high TF and release NETs carrying active TF (Skendros et al., 2020). In the culprit artery of acute myocardial infarction, NETs have also been shown to be vital for active TF delivery (Stakos et al., 2015). These studies suggest that delivery of TF by NETs is a potential mechanism of TF externalization.

Considering the critical role of platelets in immunothrombosis (Frantzeskaki et al., 2017) and the finding that activated platelets were able to stimulate neutrophils for NET release (Caudrillier et al., 2012), a platelet-NET-TF axis as a potential mechanism for immunothrombosis and subsequent sepsis-induced lung injury is indicated.

Our studies showed that the circulating microenvironment of patients with sepsis/sepsis-induced ARDS could promote the expression of TF and its exposure on the surface of neutrophils. In addition, we were able to upregulate intracellular TF expression *in vitro* when culturing PMNs with plasma from sepsis patients and sepsis ARDS. We found that the inflammatory mediator thrombin could induce circulating TF produced by neutrophils. Although a variety of cytokines have been reported as TF inducers, such as fibrin (Contrino et al., 1997), TNFα (Nestoridi et al., 2005), heme (Setty et al., 2008), and IL-6 (Bester and Pretorius, 2016), further studies are needed to determine their role in septic lung injury.

Additionally, we found that TF exposed to NETs of ARDS patients is functional and can induce thrombin generation, which in turn activates resting platelets and subsequently increases TF-enriched NETs. The activation of platelets by thrombin depends on PAR-1 cell signal transduction, as shown by the attenuation of platelet activation by PAR-1 antagonism. NETs play a key role in protein epitope function, and we found that NET inhibition by DNase I inhibited the production of TF-dependent thrombin. Correspondingly, treatment with recombinant human DNase I significantly improved the outcome of thrombosis events in mice with septic lung injury.

As described above, previous studies have suggested that activated platelet-neutrophil interactions could induce NET release in a variety of diseases, except for sepsis-induced lung injury. In this study, we found that both activated platelets obtained from ARDS and platelets activated by plasma from ARDS patients could induce TF-enriched NET formation in neutrophils from healthy donors. Elevated thrombin levels were found in ARDS patients, and thrombin inhibitors or PAR-1 blockers significantly reduced platelet-induced NET production, indicating that thrombin may play a critical role in this process. There are some limitations of this study, Firstly, we didn’t quantify *in situ* thrombosis in the treated versus untreated sepsis-ARDS mouse lung tissue. Future researches are needed to verify the direct role of immunothrombosis in progression of sepsis induced lung injury. Secondly, the surgery type of the patients enrolled in the study were different, these maybe one of the factors influence the results.

**Figure 7:**
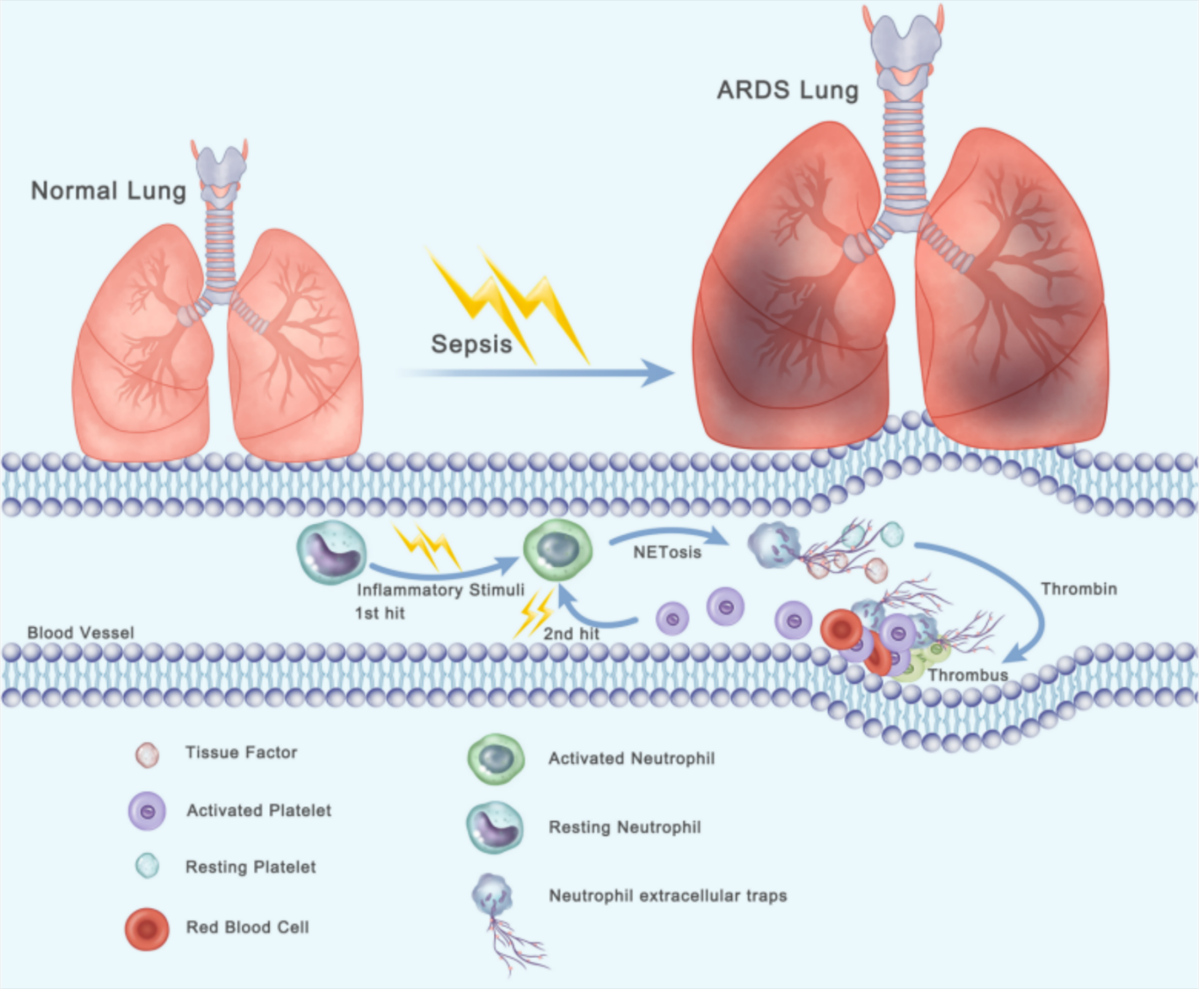
Two-hit procedures of thrombosis formation initiated by NETs and activated platelets lead to the progression of ARDS. When sepsis-mediated ARDS occurs, neutrophils are activated and then form TF-enriched neutrophil extracellular traps in pulmonary blood vessels (first step), resulting in thrombin generation. Platelets are activated by thrombin and then interact with neutrophils, which lead to formation of TF-enriched NET (second step).Thereby creating a vicious cycle that causes massive thrombosis formation.

In summary, we identified a thrombin–platelet–NET loop that contributes to immunothrombosis in sepsis-related lung injury. When sepsis-mediated ARDS occurs, neutrophils are activated and then form TF-enriched neutrophil extracellular traps in pulmonary blood vessels (first step), resulting in thrombin generation. Platelets are activated by thrombin and then interact with neutrophils, which lead to formation of TF-enriched NET (second step). all the factors thereby creating a vicious cycle that causes massive thrombosis formation (**Figure 7**). The observation that NETs are involved in TF function and thrombus stability may trigger further research on new therapeutic targets for sepsis-induced lung injury.

## Data Availability Statement

The raw data supporting the conclusions of this article will be made available by the authors, without undue reservation.

## Ethics Statement

The studies involving human participants were reviewed and approved by Research Ethical Committee of Fudan University Shanghai Cancer Center (license number: 20180109-04). The patients/participants provided their written informed consent to participate in this study. The animal study was reviewed and approved by Institutional Animal Care and Use Committee of the Fudan University.

## Author Contributions

(I) Conception and design: HZ, WC, and CM. (II) Collection and assembly of data: MQ and YY. (III) Data analysis and interpretation: HZ. (IV) Manuscript writing: All authors. All authors contributed to the article and approved the submitted version.

## Funding

National Key Research and Development Program of China (NO. 2020YFC2008400, 2020YFC2008403); National Natural Science Foundation of China (NO. 81873948, 81871591) Clinical Research Plan of SHDC (NO. SHDC2020CR4064, SHDC2020CR1005A, SHDC12018105); Key Technology and Development Program of Shanghai (NO.17411963400) Talent Program of Fudan University (JIF159607), Shanghai Sailing program (21YF1406800); Natural Science Foundation of Shanghai (21ZR1413400).

## Conflict of Interest

The authors declare that the research was conducted in the absence of any commercial or financial relationships that could be construed as a potential conflict of interest.

## Supplementary Material

Supplementary Figure 1 NET degradation protects mice against sepsis-induced lung injury. Neutrophil depletion was induced with CI-amidine, anti-Ly6G antibodies or DNase in ARDS mice. Plasma levels of Cf DNA **(A)**, MPO-DNA complexes **(B)**, and MPO **(C)** after normal saline, CI-amidine, anti-Ly6G antibodies or DNase stimulation for 6 h in control, sham or ARDS mice (n=6). **(D)** Western blots for CitH3 expression in control or ARDS mouse peripheral blood neutrophils treated with saline, CI-amidine or DNase. GAPDH was used as an internal reference. **(E)** Comparison of NETs in control, Sham, and ARDS mice. Immunofluorescence of lung tissue in red (MPO), green (CitH3), and blue (DAPI) in different groups. **(F)** IHC staining analysis of MPO^+^Ly6G^+^cell percent. **(G)** Mouse Lung injury score. **(H)** IHC staining analysis of CitH3^+^MPO^+^cell percent.

Supplementary Figure 2 NET inhibition in protecting against sepsis-induced lung injury. IHC stained NETs with neutrophil elastase (NE) and NE inhibitor to detect NET formation in different groups. Additionally, MPO activity was assessed in each group. **(A)** Immunofluorescence of NETs from mouse blood in red (MPO), green (NE), and blue (DAPI) in different groups. **(B)** The percentage of NETs releasing cells in each group. **(C)** MPO activity assay in different groups.

Supplementary Figure 3 Activation status of platelets from different patients and found that platelets from ARDS patients showed increased levels of surface activation markers compared to platelets from sepsis patients or healthy controls.Fluorescence-activated cell sorting analysis of Annexin V on platelets of HC platelets **(A)**, sepsis patients **(B)**, and ARDS patients **(C)**.

Supplementary Figure 4 Activation status of platelets from different patients and found that platelets from ARDS patients showed increased levels of surface activation markers compared to platelets from sepsis patients or healthy controls.Fluorescence-activated cell sorting analysis of CD62P on platelets of HC platelets **(A)**, sepsis patients **(B)**, and ARDS patients **(C)**.

Supplementary Figure 5 Platelet/neutrophil aggregates (CD61+CD11b+) in PMNs isolated from ARDS patients compared to sepsis patients or healthy controls. Platelet/neutrophil aggregates observed as double-positive CD61/CD11b per 10000 CD11b-positive events with fluorescence-activated cell sorting analysis in polymorphonuclear neutrophils isolated from blood of HC **(A)**, sepsis **(B)**, and ARDS patients **(C)**.
