# Supplementary figures and images for: Tissue Factor-Enriched Neutrophil Extracellular Traps Promote Immunothrombosis and Disease Progression in Sepsis-Induced Lung Injury
Source: Front Cell Infect Microbiol. 2021 Jul 14;11:677902. doi: 10.3389/fcimb.2021.677902 (PMC8317465; doi:10.3389/fcimb.2021.677902)

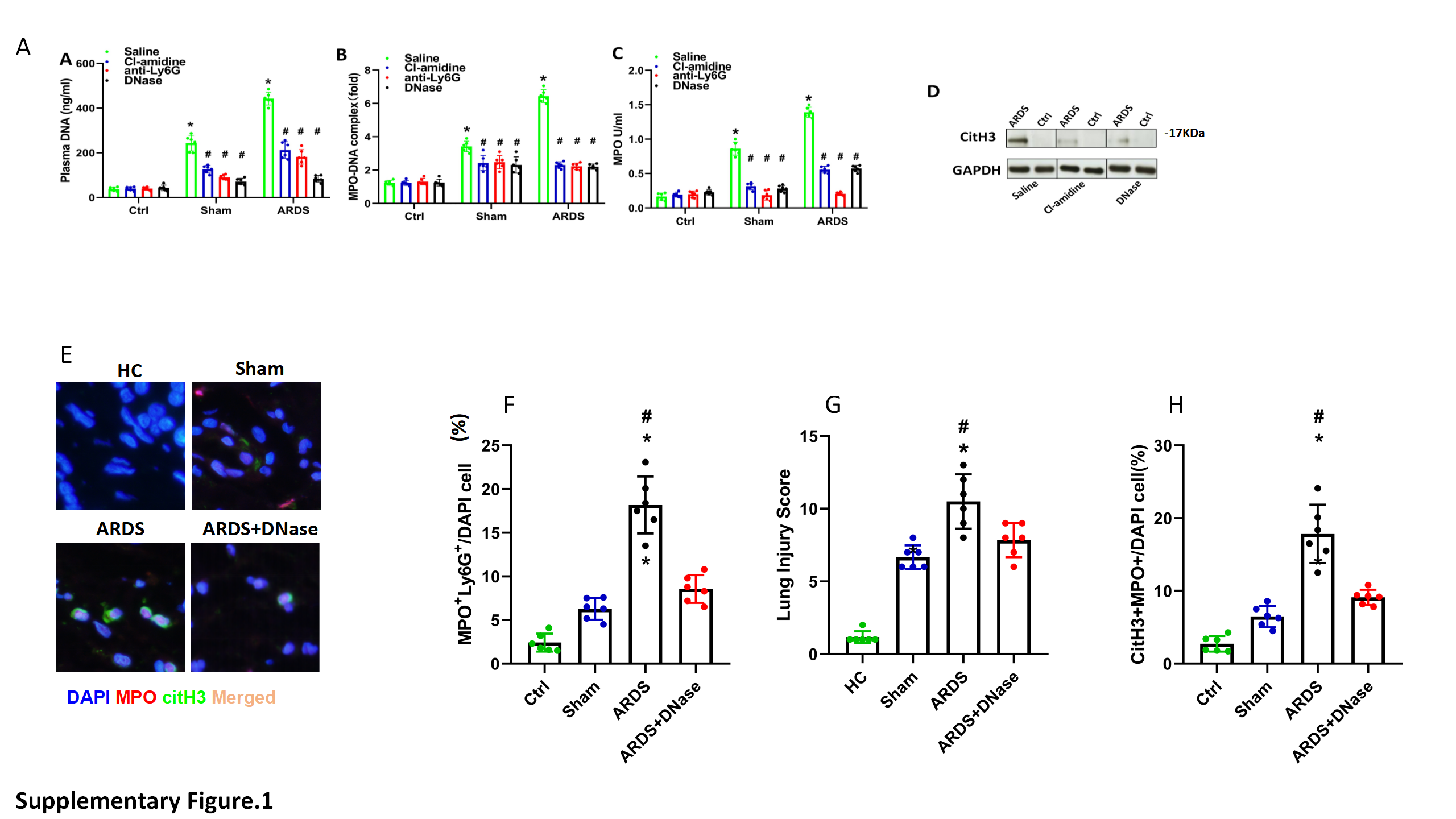

Supplement: Supplementary Figure 1 — NET degradation protects mice against sepsis-induced lung injury. Neutrophil depletion was induced with CI-amidine, anti-Ly6G antibodies or DNase in ARDS mice. Plasma levels of Cf DNA (A), MPO-DNA complexes (B), and MPO (C) after normal saline, CI-amidine, anti-Ly6G antibodies or DNase stimulation for 6 h in control, sham or ARDS mice (n=6). (D) Western blots for CitH3 expression in control or ARDS mouse peripheral blood neutrophils treated with saline, CI-amidine or DNase. GAPDH was used as an internal reference. (E) Comparison of NETs in control, Sham, and ARDS mice. Immunofluorescence of lung tissue in red (MPO), green (CitH3), and blue (DAPI) in different groups. (F) IHC staining analysis of MPO+Ly6G+cell percent. (G) Mouse Lung injury score. (H) IHC staining analysis of CitH3+MPO+cell percent. [file Image_1.tif]

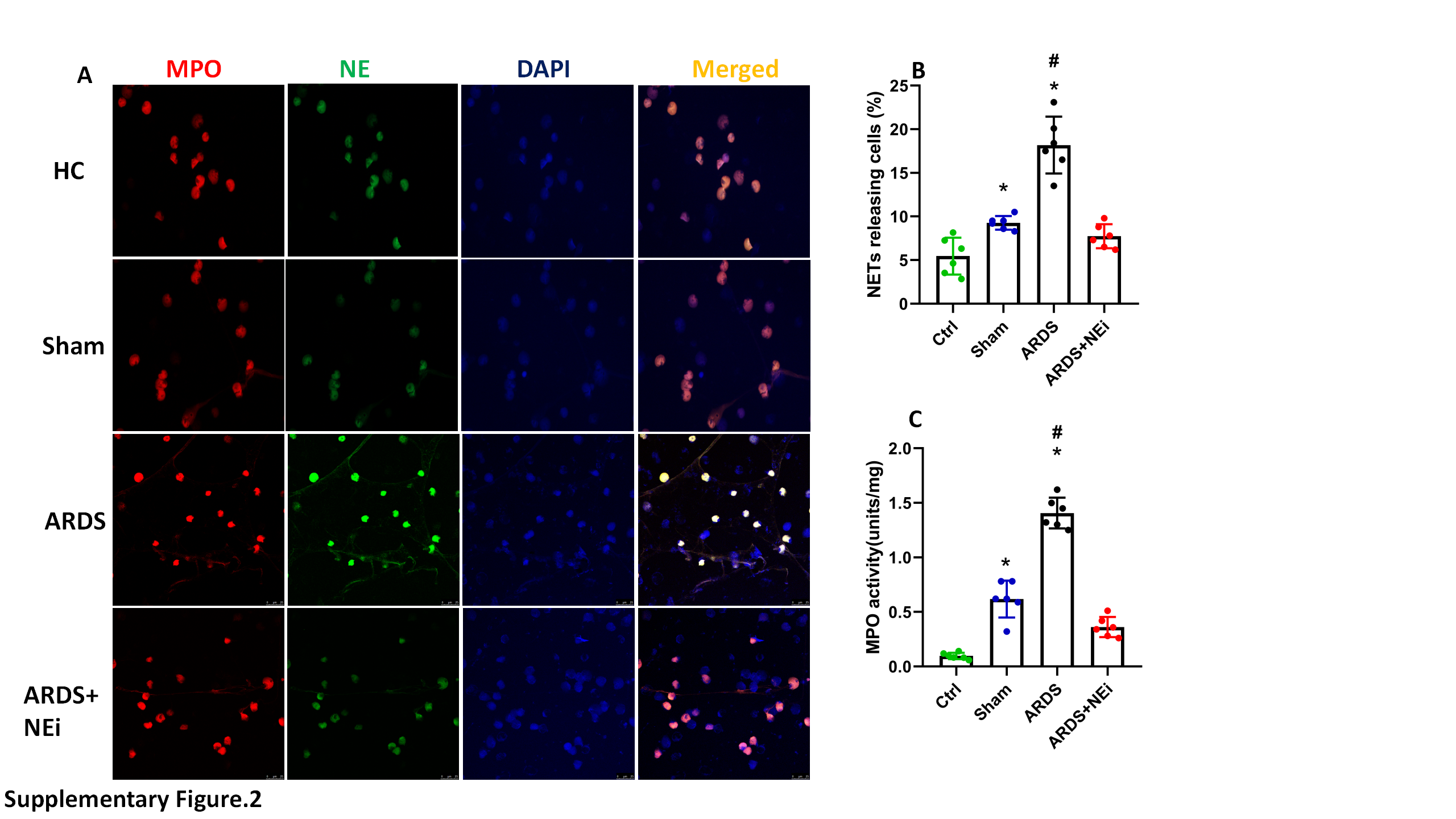

Supplement: Supplementary Figure 2 — NET inhibition in protecting against sepsis-induced lung injury. IHC stained NETs with neutrophil elastase (NE) and NE inhibitor to detect NET formation in different groups. Additionally, MPO activity was assessed in each group. (A) Immunofluorescence of NETs from mouse blood in red (MPO), green (NE), and blue (DAPI) in different groups. (B) The percentage of NETs releasing cells in each group. (C) MPO activity assay in different groups. [file Image_2.tif]

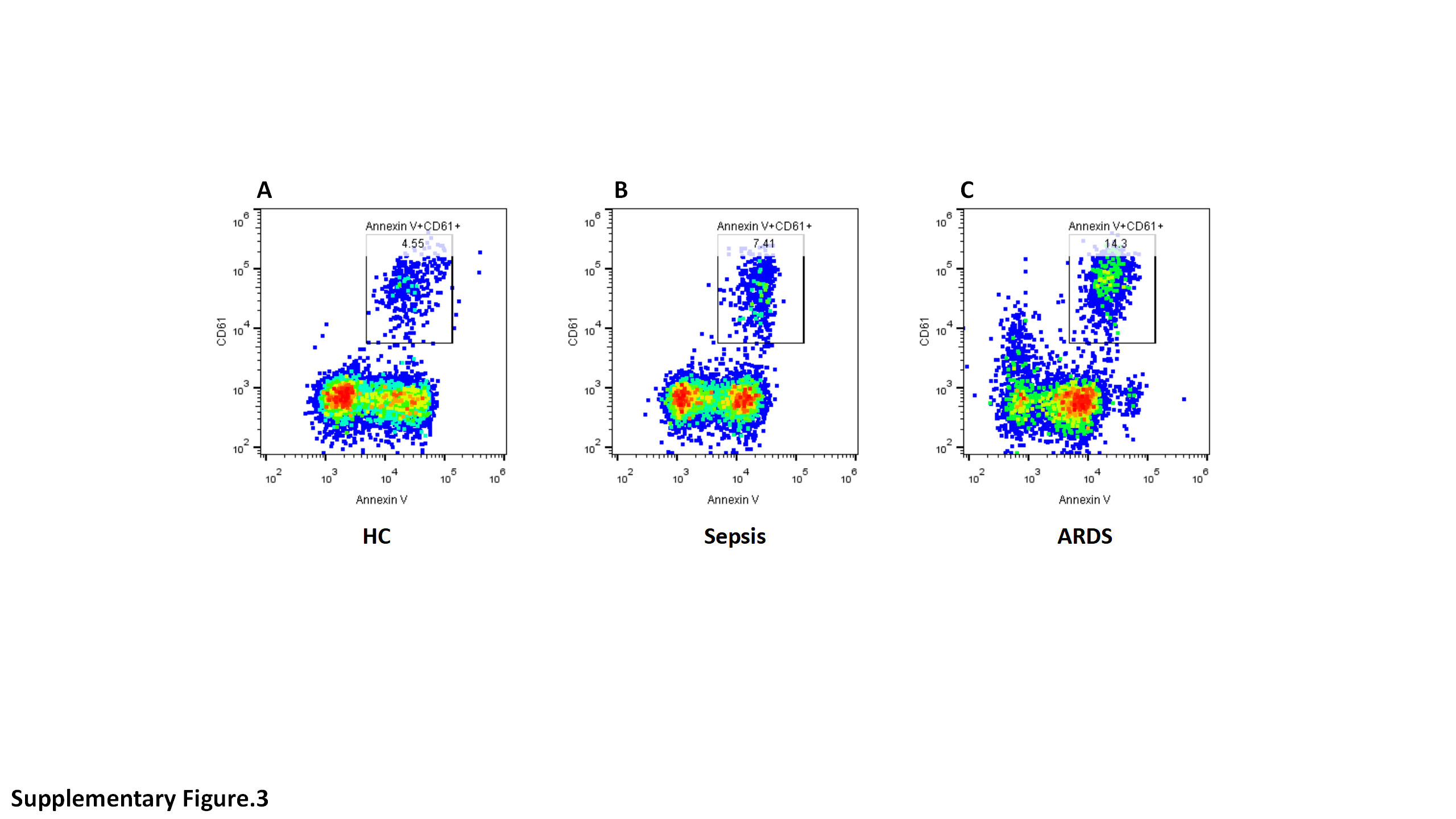

Supplement: Supplementary Figure 3 — Activation status of platelets from different patients and found that platelets from ARDS patients showed increased levels of surface activation markers compared to platelets from sepsis patients or healthy controls.Fluorescence-activated cell sorting analysis of Annexin V on platelets of HC platelets (A), sepsis patients (B), and ARDS patients (C). [file Image_3.tif]

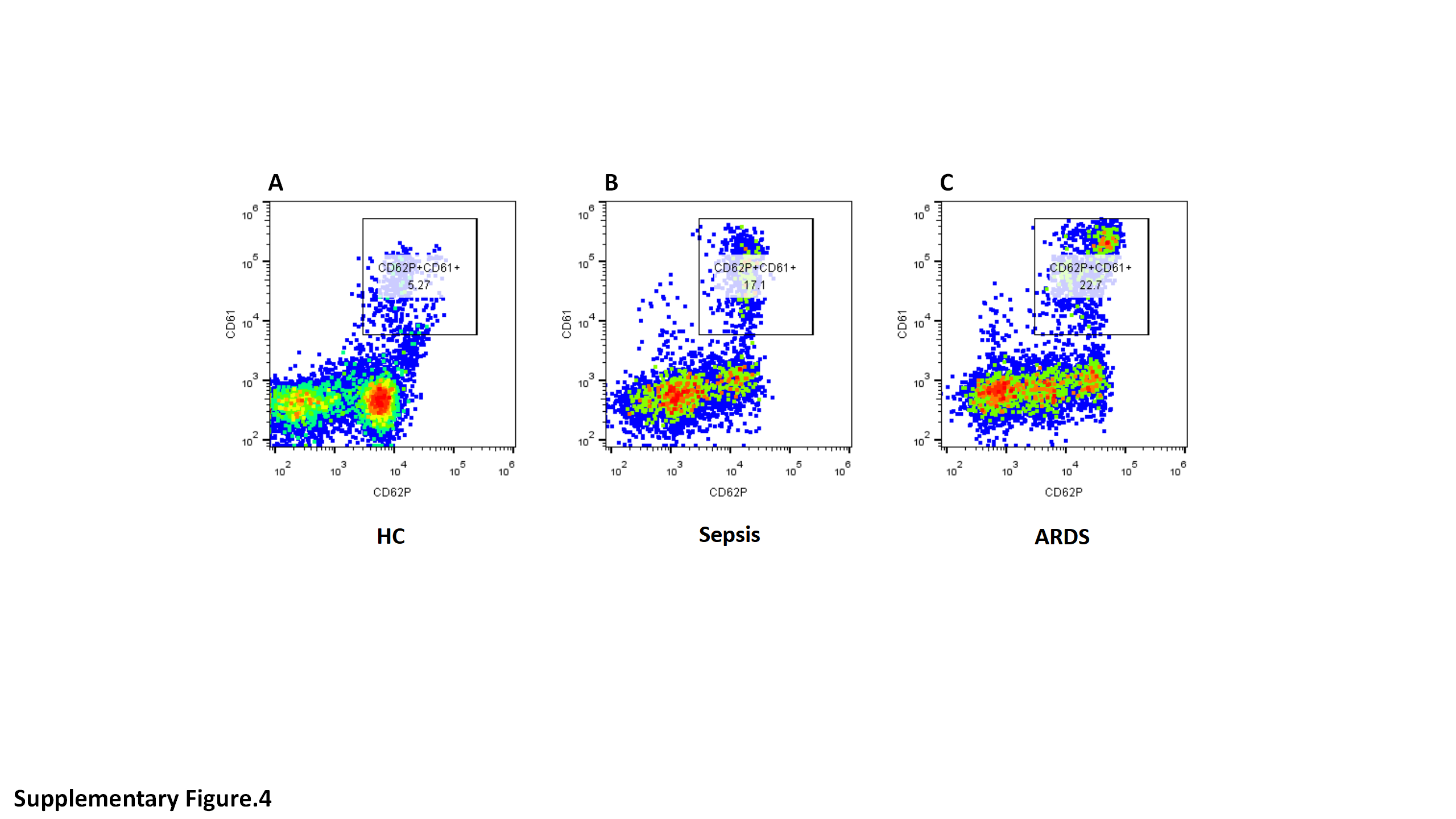

Supplement: Supplementary Figure 4 — Activation status of platelets from different patients and found that platelets from ARDS patients showed increased levels of surface activation markers compared to platelets from sepsis patients or healthy controls.Fluorescence-activated cell sorting analysis of CD62P on platelets of HC platelets (A), sepsis patients (B), and ARDS patients (C). [file Image_4.tif]

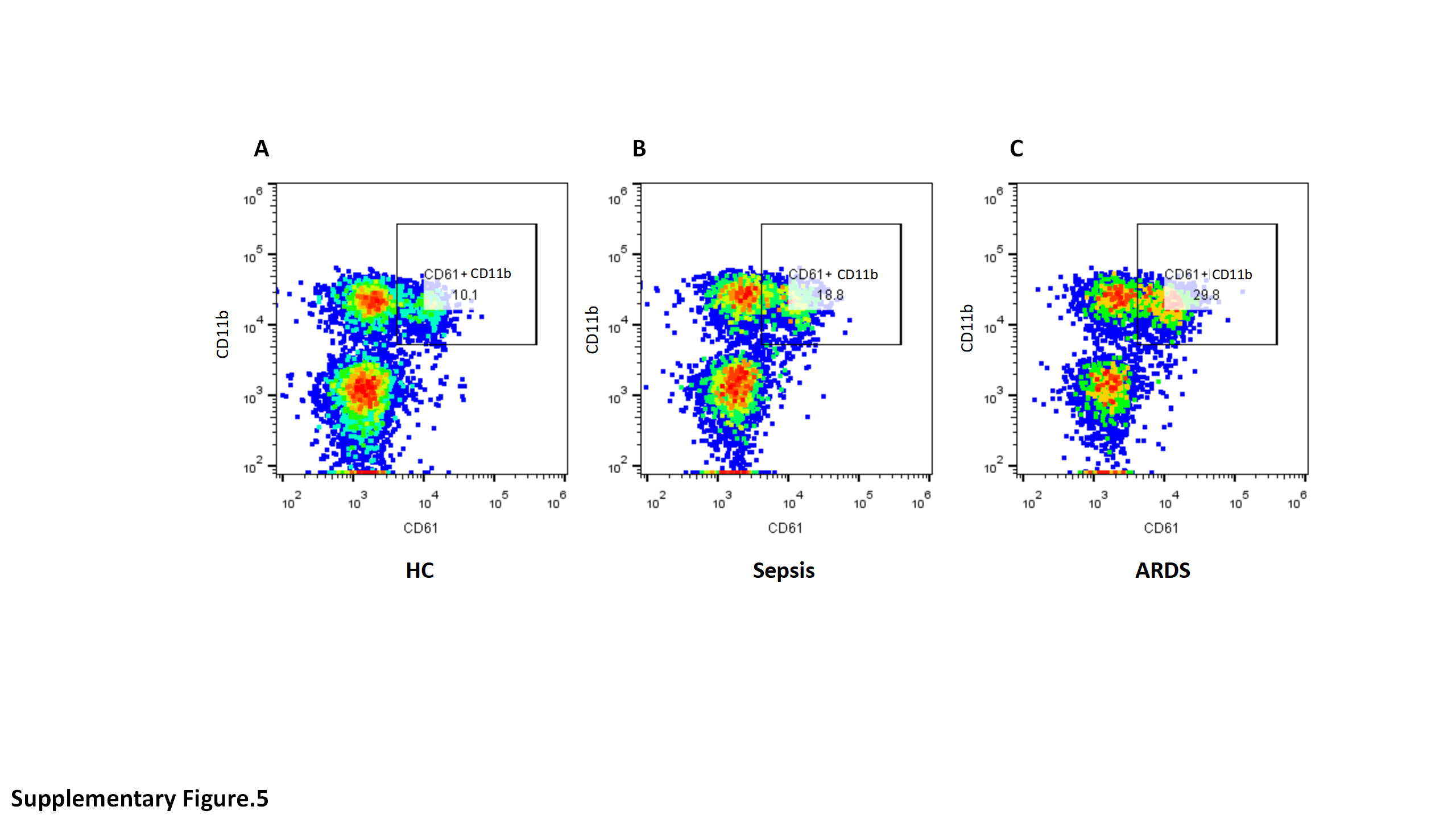

Supplement: Supplementary Figure 5 — Platelet/neutrophil aggregates (CD61+CD11b+) in PMNs isolated from ARDS patients compared to sepsis patients or healthy controls. Platelet/neutrophil aggregates observed as double-positive CD61/CD11b per 10000 CD11b-positive events with fluorescence-activated cell sorting analysis in polymorphonuclear neutrophils isolated from blood of HC (A), sepsis (B), and ARDS patients (C). [file Image_5.tif]
